# Cervical cancer in women living in South Africa: a record linkage study of the National Health Laboratory Service and the National Cancer Registry

**DOI:** 10.3332/ecancer.2022.1348

**Published:** 2022-01-27

**Authors:** Tafadzwa Dhokotera, Serra Asangbeh, Julia Bohlius, Elvira Singh, Matthias Egger, Eliane Rohner, Jabulani Ncayiyana, Gary M Clifford, Victor Olago, Mazvita Sengayi-Muchengeti

**Affiliations:** 1 National Cancer Registry, National Health Laboratory Science, 1 Modderfontein Road, Sandringham, Johannesburg 2192, South Africa; 2 Graduate School for Cellular and Biomedical Sciences, University of Bern, Uni Mittelstrasse, Mittelstrasse 43, CH-3012 Bern, Switzerland; 3 Swiss Tropical and Public Health Institute, Kreuzstrasse 2, 4123 Allschwil, Basel Land, Switzerland; 4 University of Basel, Peterspl. 1, 4001 Basel, Switzerland; 5 Institute of Social and Preventive Medicine, University of Bern, Mittelstrasse 43, CH-3012 Bern, Switzerland; 6 School of Public Health, University of the Witwatersrand, 27 St Andrews Rd, Parktown, Johannesburg 2193, South Africa; 7 Population Health Sciences, Bristol Medical School, University of Bristol, Beacon House, Queens Road, Bristol BS8 1QU, UK; 8 Centre for Infectious Disease Epidemiology and Research (CIDER), School of Public Health and Family Medicine, University of Cape Town, Falmouth Rd, Observatory, Cape Town 7925, South Africa; 9 Department of Public Health Medicine, School of Nursing and Public Health, College of Health Sciences, University of KwaZulu-Natal, Howard College Campus, George Campbell Building, 2nd Floor, Rm 226, 238 Mazisi Kunene Rd, Glenwood, Durban 4041, South Africa; 10 Infections and Cancer Epidemiology Group, International Agency for Research on Cancer, (IARC/WHO), 150 cours Albert Thomas, 69372 Lyon, Cedex 08, France; 11 South African DSI-NRF Centre of Excellence in Epidemiological Modelling and Analysis (SACEMA), Stellenbosch University, 19 Jonkershoek Road, Stellenbosch 7600, South Africa

**Keywords:** cervical cancer, HIV, South Africa, epidemiology

## Abstract

**Introduction:**

In countries with high HIV prevalence, it is important to understand the cervical cancer (CC) patterns by HIV status to ensure targeted prevention measures. We aimed to determine the factors associated with CC compared to non-infection related cancer in women living in South Africa.

**Methods:**

This was a cross-sectional study of women aged 15 years and older diagnosed with CC and non-infection related cancer in the South African public health sector from 2004 to 2014. The National Cancer Registry provided data on cancer, whilst HIV status was determined from routinely collected HIV related data from the National Health Laboratory Service. We explored the association of HIV infection, age, ethnicity and calendar period with CC compared to non-infection related cancer.

**Results:**

From 2004 to 2014, 49,599 women were diagnosed with CC, whilst 78,687 women had non-infection related cancer. About 40% (*n* = 20,063) of those with CC and 28% (*n* = 5,667) of those with non-infection related cancer had a known HIV status. The median age at CC diagnosis was 44 years (interquartile range (IQR): 37–52) and 54 years (IQR: 46–64) for HIV positive and negative women, respectively, and for non-infection related cancer, 45 years (IQR: 47–55) and 56 years (IQR: 47–66) for HIV negative and positive women, respectively. Diagnosis of CC was associated with HIV positivity, Black ethnicity, earlier calendar period (2004–2006) and the ages 30–49 years. In comparison with Black women, the odds of CC were 44% less in Coloured women, 50% less in Asian women and 51% less in White women.

**Conclusions:**

HIV positive women presented a decade earlier with CC compared to HIV negative women. A large proportion of women with CC were unaware of their HIV status with a disproportionate burden of CC in Black women. We recommend women attending CC screening facilities to be offered HIV testing so that recommendations for their follow-up visits are given according to their HIV status.

## Introduction

Cervical cancer (CC) is the second most common cancer and the leading cause of cancer-related deaths amongst women in South Africa [1]. The burden of CC is highest in women living with HIV. In 2018, approximately 63% of women with CC were HIV positive, with 53% of CC cases attributable to HIV in South Africa [2]. CC can be prevented through Human Papilloma Virus (HPV) vaccination, screening and treatment of precancerous lesions. Consequently, the World Health Organization has called for the elimination of CC, the first cancer ever to be targeted for elimination [3]. The implementation of prevention measures should be evidence-based to ensure efficient allocation of resources and maximum public health benefits in resource-limited settings [4].

Age has long been considered a significant parameter in the secondary prevention of CC with the optimum age for CC screening varying from about 20 to 30 years in high income countries [5]. The recommended starting age for CC screening in South African women is 30 years for the general population and at HIV diagnosis, irrespective of age, for HIV positive women [6]. Black women have a higher burden of HIV infection when compared to other ethnicities in South Africa and the burden of CC is also high in this demographic group regardless of HIV status [7, 8]. In South Africa, data on CC are available in the general population; however, data are limited for women living with HIV at the national level. A high proportion of CC has been attributed to HIV with younger women living with HIV increasingly presenting with CC. Yet, an estimated 48% of women aged 15 years and older are unaware of their HIV positive status [2, 8]. As a result, HIV positive women who are unaware of their status present a gap in the risk stratified cervical screening efforts.

To ensure women follow the recommended screening guidelines specific to their risk profile, information on CC burden by age, ethnicity and HIV status (positive, negative and unknown) is crucial. The South African Match (SAM) study provides a unique opportunity to achieve such an objective. The SAM database contains linked routinely collected HIV and cancer records of clients seeking care in South African public health care facilities [9]. A cross-sectional sub-study of the SAM study assessed the burden of cancers attributable to HIV [10]. The current short report builds on this analysis to determine the association of HIV infection, age, ethnicity and calendar period with CC compared to non-infection related cancer.

## Methods

### Study population

This was a cross-sectional study of women aged 15 years and older diagnosed with CC or non-infection related cancer in the South African public health sector from January 2004 to December 2014 as reported to the National Cancer Registry (NCR) of South Africa.

### Data sources

We linked cancer data from the NCR to routinely collected HIV related data from the National Health Laboratory Service (NHLS). The NCR collects data from both private and public laboratories on pathology-confirmed cancer diagnoses [11, 12]. NHLS HIV data are collected through a network of laboratories that provides pathology services to the public health sector of South Africa. Data from the NHLS represent 80% of the South African population. We excluded data from the private sector in the NCR dataset as we assumed that women who had their cancer diagnosed in the public sector would potentially get their HIV care in the private sector as well. From the linkage, only 0.08% (*n* = 6/7,550) of CC cases diagnosed in the private sector matched to the NHLS HIV data.

### Variables and data management

We defined CC as per International Classification of Disease for Oncology Volume 3 classification (topography C53) [13]. Since we were unable to select an undiseased comparison group, we selected controls from women with non-infection related cancers in the NCR database. This has been done before by the Johannesburg Case–Control Study [14]. The following cancers as per topography were used as the comparison group: Breast (C50), Endometrium (C54-55), Colon (C18-20), Oesophagus (C15), Ovaries (C56), Mucinous (C56 & 8470/3, 8471/3, 8472/3, 8473/3), Clear cell (C56 & 8310/3), Lung Cancer (C33-34), Myeloma (C90), Pancreas (C25), Myeloid Leukaemia (ICD-10 C92), Placenta (C58.9), Melanoma (C43), Bone (C40-41), Larynx (C32), Thyroid (C73), Kidney (C64), Brain (C71), Peritoneum and retroperitoneum (C48), Endocrine gland (C75), Small Intestine (C17), Meninges (C70), Thymus (C37), Fallopian tube (C57.0), Central Nervous System (C72) and Peripheral nerves & Autonomic Nervous System (C47). HIV status was determined from HIV diagnostic and monitoring tests. Diagnostic tests included enzyme linked immunosorbent assays, western blots and rapid tests. HIV monitoring tests included cluster of differentiation 4 (CD4) cell counts and HIV RNA viral loads. Demographic characteristics like age, ethnicity and year of cancer diagnosis were obtained from the cancer data. Age was categorised into 5-year age groups, and ethnicity was grouped as per the Statistics South Africa groupings, i.e., Asian, Black, Coloured (mixed race) and White. Year of cancer diagnosis was categorised into the following calendar periods: 2004–2006, 2007–2010 and 2011–2014.

### Data Analysis

We described our study population by HIV status, age, ethnicity, year of cancer diagnosis stratified by CC diagnosis versus non-infection related cancer. We performed a test of proportions to determine any differences between categories. We treated age as a continuous variable and used Kruskal–Wallis to test for differences in median age at CC diagnosis by HIV status. We used logistic regression to determine the factors associated with CC compared to non-infection related cancer in women living in South Africa. All analyses were done using Stata (version 16, College Station, TX, USA).

## Results

From 2004 to 2014, out of 128,286 women who met our eligibility criteria, 49,599 women aged 15 years and older were diagnosed with CC. A total of 40,190 women (31.3%) had a known HIV status. Amongst women with CC, 20,063 (40.5%) had a known HIV status. Of women with CC and a known HIV status, 58.1% (11,656 of 20,063) were assigned an HIV positive result. The percentage with an HIV result increased from 15% (*n* = 633/3,981) of all women with CC diagnosed in 2004 to 50% (*n* = 2,128/4,288) in 2014. We had a total of 78,687 non-infection related cancer. About 28% (5,667/20,127) of those with non-infection related cancer had a known HIV status (Table 1). The percentage with an HIV result for this group increased from 8.2% (*n* = 137/6,286) in 2004 to 28.4% (*n* = 2,661/7,492) in 2014. The median age of women diagnosed with CC was 51 years (interquartile range (IQR): 42–62), whilst the median age of women diagnosed with non-infection related cancer was 58 years (IQR: 47–68).

**Figure 1.**
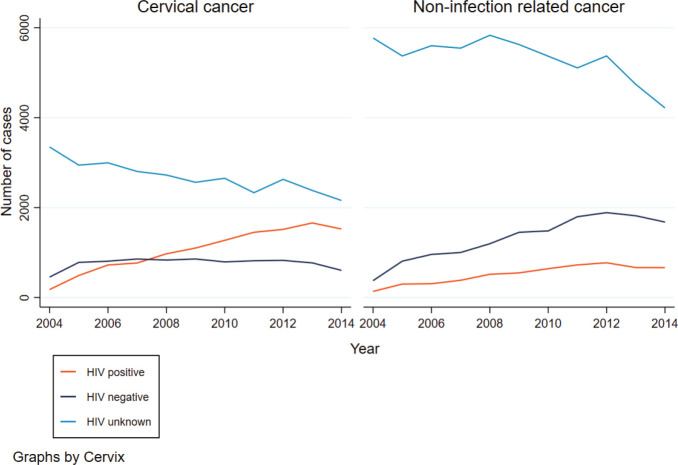
Trends in the frequency of CC and non-infection related cancer by HIV status.

From 2004 to 2014, the number of women diagnosed with CC and HIV increased. In 2008, the number of women with CC and diagnosed with HIV surpassed that of HIV negative women with CC (Figure 1). A decline in the number of cases was seen in 2014. For women with a non-infection related cancer, the number of women increased for both HIV positive and negative women, with more cases observed in HIV negative women whilst a decline was observed for those who had an unknown HIV status. The age distribution between HIV positive and negative women with CC differed. The median age at CC diagnosis for HIV negative women was 54 years (IQR: 46–64) and 44 years (IQR: 37–52) for HIV positive women (*p*-value < 0.001). More than half of all HIV positive women were diagnosed with CC between the ages of 35 and 49 years (52%, *n* = 6,010), and only 11% (*n* = 1,326) of CCs were diagnosed in women aged 60 years or older. In contrast, among HIV negative women, 31% (*n* = 2,565) were diagnosed with CC at ages of 35–49 years, and another 34% (*n* = 2,888) at ages of 60 years or older. For women with an unknown HIV status, the pattern was similar for HIV negative women with 35% (*n* = 10,379) diagnosed with CC at ages of 60 years and older.

**Figure 2.**
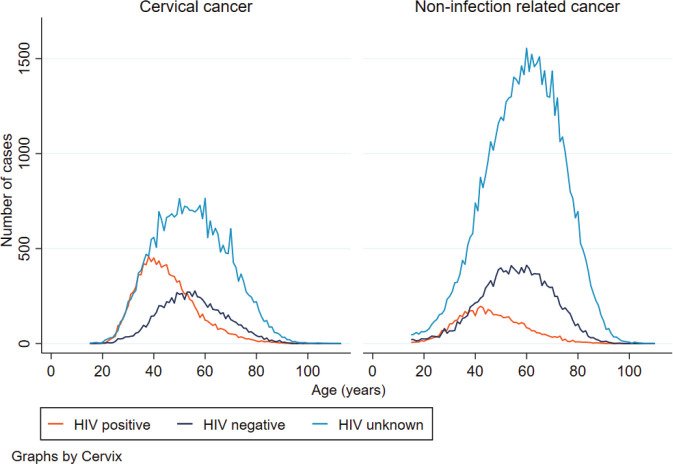
Distribution of age at CC and non-infection related cancer diagnosis by HIV status.

Similarly, amongst women with non-infection related cancer, the age distribution between HIV positive and negative women differed. The median age at non-infection related cancer diagnosis was 45 years (IQR: 37–55) for HIV positive women and 56 years (IQR: 47–66) for HIV negative women. About 44% (*n* = 2,487) of women diagnosed with HIV and non-infection related cancer were in the 35–49 years age group whilst for HIV negative women with non-infection related cancer, only 26% (*n* = 3,705) of cases were observed between the ages of 35 and 49 years and a further 41% (*n* = 5,896) in those 60 years and older. The age distributions were similar for CC and non-infection related cancer. However, for women living with HIV and diagnosed with CC, there was a steeper increase and more cases from the age 20 to about 40 years compared to women living with HIV and diagnosed with non-infection related cancer in the same age range (Figure 2).

**Figure 3.**
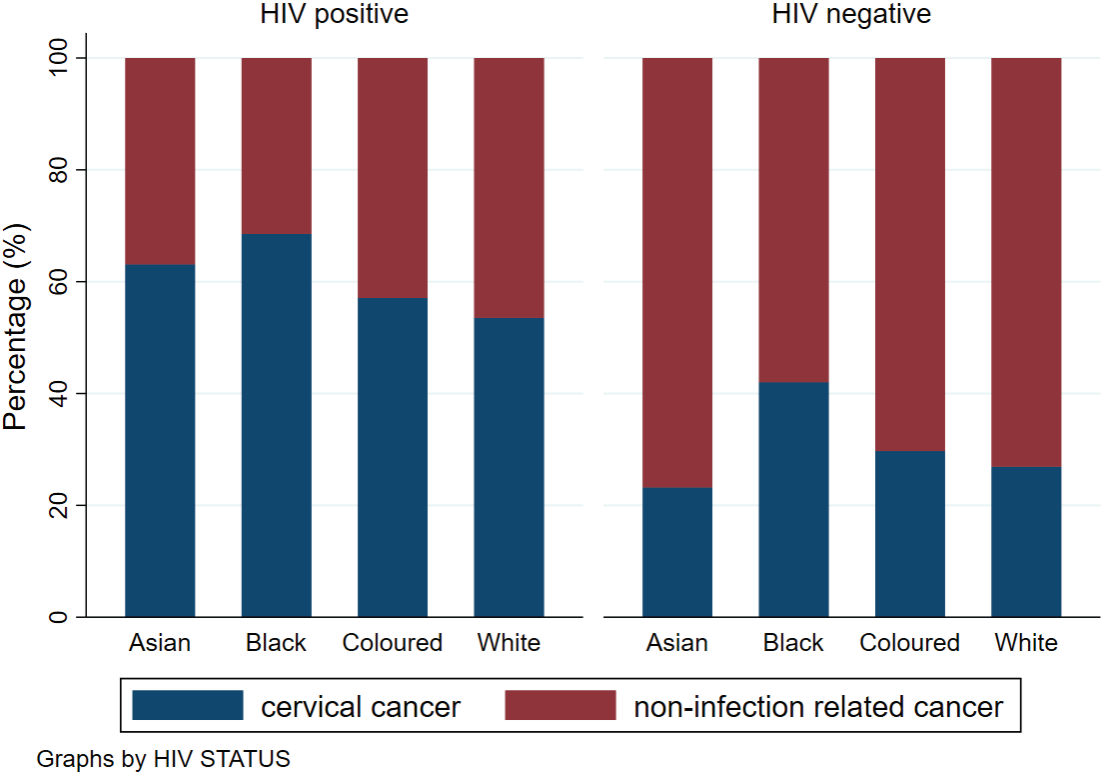
Percentage of CC and non-infection related cancer by HIV status and ethnicity.

Across all ethnicities, the percentage of HIV positivity was higher amongst those with CC compared to those without CC (Figure 3). However, when we determined the association between CC and ethnicity, the odds of CC were 50% less in Asian women (Odds ratio (OR): 0.50 (95% confidence interval (CI): 0.41–0.61)), 44% less in Coloured women (OR: 0.56 (95% CI: 0.52–0.59)) and 51% less in White women (OR: 0.49 (95% CI: 0.46–0.53)) (Table 2). Compared to the calendar period 2004–2006, the odds of CC diagnosis were lower in the subsequent calendar periods with an OR of 0.75 (95% CI: 0.70–0.80) for the period 2007–2010 and an OR of 0.64 (95% CI: 0.60–0.68) observed in the later calendar periods. Regarding age, there was no difference observed in the odds of CC amongst women aged 30–34 years and those between the ages of 40 and 49 years compared to women aged 35–39 years.

## Discussion

The results of this cross-sectional analysis of national South African cancer registry data show that women diagnosed with CC and with HIV were a decade younger than women with CC who were HIV negative. Diagnosis of CC was associated with being Black, HIV positivity, early calendar period (2004–2006) and the ages 30–49 years. The percentage of women diagnosed with CC with a known HIV status increased from 15% in 2004 to approximately 50% in 2014, and over half of the women diagnosed with a CC had an unknown HIV status.

Our study’s main limitation was the high percentage of CC diagnoses with an unknown HIV status. Given that we assigned HIV status from routinely collected HIV data, we might have missed individuals tested for HIV through point of care tests not recorded by the NHLS as well as those tested by the private sector. However, it essential to note that we excluded CC cases diagnosed in the private sector. Only 0.08% of CC cases in the private sector were assigned an HIV results. We then assumed that those CC cases diagnosed in the private sector would have their HIV tests also done in the private sector (and hence not recorded in NHLS). As a result, we might have introduced some selection bias as use of the private sector is largely associated with better socio-economic status. Identifiable data of the national population of women to link to the CC cases as well as HIV data were not available; therefore, our assessment on ethnicity might not reflect the true distribution in the South African population. However, we still believe that even though we had no access to these population estimates, our study provides useful information by demonstrating that a large proportion of women diagnosed with CC are unaware of their HIV status and the disparities in CC by ethnicity.

We and others observed a similar pattern of earlier presentation of CC in women living with HIV than among HIV negative women [15–19]. Between 2008 and 2010, a study used NHLS data to determine the age at diagnosis of invasive and pre-invasive CC in the South African province of Limpopo [19]. In that study, the mean age at invasive CC diagnosis was 41.3 years (standard deviation (SD): 9.7) for HIV positive, and 59.1 years (SD: 14.7) for HIV negative women [19]. A nationwide study of the NHLS cervical cancer dataset from 2014 to 2016 established that the peak age at CC diagnosis was 36–40 years amongst HIV positive women and >60 years in HIV negative women [20]. In Zambia, the median age at CC diagnosis was 40 years for HIV negative women and 35 years for HIV positive women [21]. The median age at CC diagnosis that we observed for women living with HIV in our study is higher compared to most studies from sub-Saharan Africa. This could likely be because of late presentation to health facilities and subsequent diagnosis at advanced stages. However, we cannot verify this with our data, as we did not have staging data.

CC was associated with HIV positivity, Black ethnicity and early calendar period (2004–2006). The association of HIV infection with CC is consistent across literature [22–24]. Regarding ethnicity, in South Africa, the prevalence of HIV is highest in Black African women at 27.8% for those aged 25 years and older [8]. This HIV prevalence is significantly higher than in Asian (0.9%), White (1.3%) and Coloured (7.8%) ethnicities. As a result, the higher odds of CC observed in Black women compared to all other ethnicities might reflect the higher burden of HIV in this population. Barriers to CC screening amongst Black women have also been implicated as potential contributing factors to the increased burden in this subpopulation. Currently in South Africa, the women are offered three free Pap smears in their lifetime of 10 year intervals with the recommended age at first screening being 30 years for the general population [6]. For women living with HIV, the recommendation is to receive screening at HIV diagnosis and every 3 years thereafter if the screening test is negative [6]. Similarly, the WHO guidelines also recommend CC screening at age of 30 years for the general population [25]. However, for women living with HIV, the recommended age is 25 years. The recommended screening method for both HIV positive and negative women is HPV DNA detection. However, in areas where cytology is used as the primary screening test, it should continue to be used until HPV DNA testing can be introduced. To work towards the CC elimination agenda, by 2030, 70% of women should be screened by age 35 years and again by age 45 years for cervical precancerous lesions using a high-performance test, and 90% of those requiring treatment have to be treated [25]. In a cross-sectional study in South Africa, they observed that the Black ethnic group was associated with a less likelihood of screening for CC [26]. Specifically, 40% of Black women reported ever having done a Pap smear compared to 91% White, 83% Coloured and 78% of Asian women [26]. The barriers to CC screening in black women in South Africa include lower socioeconomic position, lack of knowledge on CC screening, cultural beliefs as well as fear and stigma [26–29].

Whilst the percentage of HIV unknown declined across the years, in 2014, the percentage of CC diagnosis with an unknown HIV status was still high. The percentage of HIV unknowns in our study is close to the estimated 45% of women aged 15 years and older in South Africa, who were unaware of their HIV status in 2012 [30]. The improvement in HIV testing we observed from 2004 is likely explained by the expansion of the HIV testing programme in South Africa, with more people gaining access to HIV testing services. According to a national, representative survey, the percentage of women ever tested for HIV increased from 33% in 2005 to 79% in 2017 [31]. In the context of CC, a case–control study demonstrated that approximately 10% of women diagnosed with CC were unaware of their HIV positive status [32]. Over time, we also observed an increase in the percentage of HIV positive CC cases. This might reflect the better longevity in people living with HIV due to improvements in and expansion of antiretroviral treatment. With reduced HIV related mortality, women with HIV may now live long enough to develop and be diagnosed with CC [33, 34]. Another possible explanation of this is the expansion of the HIV testing programme in South Africa in years that are more recent.

The early presentation of CC in women living with HIV is consistent with available literature. Therefore, early and frequent screening for cervical pre-cancer amongst women living with HIV should continue to be strengthened. In addition to this, South African women should be encouraged to test for HIV, especially high risk populations such as Black women aged 20–34 years [5] to identify HIV positive women who are at a higher risk for CC development. We recommend women attending CC screening facilities to be offered HIV testing so that recommendations for their follow-up visits are given according to their HIV status. All these measures aim to prevent HIV positive women being assigned the general population’s screening recommendations, which recommend initiating screening at the age of 30 years and less frequently thereafter if tested negative. If women are unaware of their HIV status, this presents a gap in the elimination of CC in South Africa. We also recommend that screening efforts be intensified in the Black population.

## Conclusion

Amongst women diagnosed with CC in South Africa, women living with HIV presented a decade earlier with CC compared to HIV negative women. A large proportion of women diagnosed with CC are unaware of their HIV status and there are disparities in CC by ethnicity specifically for Black women. Given the high HIV burden in the country and in women diagnosed with CC, early and frequent screening for cervical pre-cancer should continue to be encouraged for women living with HIV. We recommend women attending CC screening facilities to be offered HIV testing so that recommendations for their follow-up visits are given according to their HIV status.

## List of abbreviations

CI, Confidence interval; HIV, Human immunodeficiency virus; HPV, Human papilloma virus; IQR, Interquartile range; NCR, National Cancer Registry; NHLS, National Health Laboratory Service; OR, Odds ratio; SAM, South African HIV Cancer Match Study.

## Conflicts of interest

The authors declare no competing interests.

## Authors’ contributions

ME, ES, MS and JB contributed towards the study design. TD contributed towards literature search, data analysis and drafting of first version of manuscript. ES and MS contributed towards data acquisition. VO contributed towards data management. All authors contributed towards data interpretation and critical comments on the first and subsequent drafts of the manuscript. All authors read and approved the final manuscript.

## Figures and Tables

**Table 1.** Characteristics of women diagnosed with CC or non-infection related cancer.

	CC		Non-infection related cancer	
	*n* = 49,599	Percentage (%)	*n* = 78,687	Percentage (%)
**Age category (years)**				
15–19	22	0.00	404	0.50
20–24	196	0.40	689	0.90
25–29	1,181	2.40	1,389	1.80
30–34	2,981	6.00	2,547	3.20
35–39	4,887	9.90	4,132	5.30
40–44	6,014	12.10	6,103	7.80
45–49	6,333	12.80	7,780	9.90
50–54	6,254	12.60	8,849	11.20
55–59	5,608	11.30	9,493	12.10
60+	14,593	29.40	35,508	45.10
Missing	1,530	3.10	1,793	2.30
Median age (interquartile range)	51 (42–62)		58 (47–68)	
**Ethnicity**				
Asian	504	1.00	2,648	3.40
Black	40,396	81.40	45,808	58.20
Coloured	3,566	7.20	12,185	15.50
White	3,160	6.40	14,830	18.80
Missing	1,973	4.00	3,216	4.10
**Cancer diagnosis year**				
2004	3,981	8.00	6,286	8.00
2005	4,217	8.50	6,482	8.20
2006	4,529	9.10	6,867	8.70
2007	4,429	8.90	6,932	8.80
2008	4,532	9.10	7,547	9.60
2009	4,522	9.10	7,626	9.70
2010	4,718	9.50	7,492	9.50
2011	4,601	9.30	7,632	9.70
2012	4,971	10.00	8,035	10.20
2013	4,812	9.70	7,227	9.20
2014	4,287	8.60	6,561	8.30
**HIV status**				
HIV negative	8,407	16.90	14,460	18.40
HIV positive	11,656	23.50	5,667	7.20
HIV unknown	29,536	59.50	58,560	74.40

**Table 2.** Factors associated with CC compared to non-infection related cancers women living in South Africa.

	Bivariate analyses	Multivariable analysis
	Odd ratio (95% CI)	Odd ratio (95% CI)
**HIV status**		
HIV negative	1	1
HIV positive	3.54 (3.39–3.69)	2.77 (2.63–2.91)
**Age category (years)**		
15–19	0.05 (0.0﻿3–0.07)	0.03 (0.01–0.07)
20–24	0.24 (0.20–0.28)	0.21 (0.16–0.27)
25–29	0.72 (0.66–0.78)	0.74 (0.64–0.85)
30–34	0.99 (0.93–1.06)	0.91 (0.82–1.01)
35–39	1	1
40–44	0.83 (0.79–0.88)	1.02 (0.93–1.11)
45–49	0.69 (0.65–0.73)	0.96 (0.88–1.05)
50–54	0.6 (0.57–0.63)	0.89 (0.81–0.97)
55–59	0.5 (0.47–0.53)	0.83 (0.76–0.91)
60+	0.35 (0.33–0.36)	0.66 (0.61–0.72)
**Ethnicity**		
Asian	0.22 (0.20–0.24)	0.5 (0.41–0.61)
Black	1	1
Coloured (mixed race)	0.33 (0.32–0.35)	0.56 (0.52–0.59)
White	0.24 (0.23–0.25)	0.49 (0.46–0.53)
**Calendar period**		
2004–2006		1 (1.00–1.00)
2007–2010	0.95 (0.92–0.98)	0.75 (0.70–0.80)
2011–2014	0.98 (0.95–1.01)	0.64 (0.60–0.68)

Multivariable logistic regression analysis adjusted for HIV status, age, ethnicity and calendar period.

95% CI, 95% confidence interval

## References

[1] Olorunfemi G, Ndlovu N, Masukume G (2018). Temporal trends in the epidemiology of cervical cancer in South Africa (1994–2012). Int J Cancer.

[2] Stelzle D, Tanaka LF, Lee KK (2021). Estimates of the global burden of cervical cancer associated with HIV. Lancet Glob Health.

[3] Das M (2021). WHO launches strategy to accelerate elimination of cervical cancer. Lancet Oncol.

[4] Baussano I, Bray F (2019). Modelling cervical cancer elimination. Lancet Pub Health.

[5] Shisana O, Zungu N, Evans M (2015). The case for expanding the definition of ‘key populations’ to include high-risk groups in the general population to improve targeted HIV prevention efforts. South African Med J.

[6] National Department of Health (2017). Cervical cancer prevention and control policy. https://www.google.co.za/search?q=The+National+Policy+on+Cervical+Cancer&rlz=1C1KAFB_enZA523ZA524&oq=The+National+Policy+on+Cervical+Cancer&aqs=chrome..69i57.1342j0j8&sourceid=chrome&ie=UTF-8.

[7] South African National Cancer Registry Cancer in South Africa 2014. http://www.nicd.ac.za/wp-content/uploads/2017/03/2014-NCR-tables-1.pdf.

[8] Simbayi LC, Zuma K, Zungu N (2019). South African National HIV prevalence, incidence, behaviour and communication survey, 2017. http://www.hsrc.ac.za/en/departments/hsc/National_HIV_Survey.

[9] Muchengeti M, Bartels L, Olago V Cohort Profile: The South African HIV Cancer Match Study (SAM) preprint.

[10] Dhokotera T, Bohlius J, Spoerri A (2019). The burden of cancers associated with HIV in the South African public health sector, 2004-2014: a record linkage study. Infect Agents Cancer.

[11] Singh E, Sengayi M, Urban M (2014). The South African national cancer registry: an update. Lancet Oncol.

[12] Singh E, Ruff P, Babb C (2015). Establishment of a cancer surveillance programme: the South African experience. Lancet Oncol.

[13] Fritz A, Percy C, Jack A (2013). International Classification of Diseases for Oncology: Third Edition.

[14] Stein L, Urban MI, O’Connell D (2008). The spectrum of human immunodeficiency virus-associated cancers in a South African black population: results from a case-control study, 1995-2004. Int J Cancer.

[15] Mpunga T, Znaor A, Uwizeye FR (2018). A case–control study of HIV infection and cancer in the era of antiretroviral therapy in R wanda. Int J Cancer.

[16] Diarra A, Botha H (2017). Invasive cervical cancer and human immunodeficiency virus (HIV) infection at Tygerberg Academic Hospital in the period 2003–2007: demographics and characteristics. South Afr J Gynaecol Oncol.

[17] Mudini W, Palefsky JM, Hale MJ (2018). Human papillomavirus genotypes in invasive cervical carcinoma in HIV-seropositive and HIV-seronegative women in Zimbabwe. J Acquir Immune Defic Syndr.

[18] Rudd P, Gorman D, Meja S (2017). Cervical cancer in southern Malawi: a prospective analysis of presentation, management and outcomes. Malawi Med J.

[19] Van Bogaert LJJ (2011). Age at diagnosis of preinvasive and invasive cervical neoplasia in south africa: HIV-positive versus HIV-negative women. Int J Gynecol Cancer.

[20] Jordaan S, Michelow P, Richter K (2016). A review of cervical cancer in South Africa: previous, current and future. Heal Care Curr Rev.

[21] Kapambwe S, Sahasrabuddhe VV, Blevins M (2015). Implementation and operational research: age distribution and determinants of invasive cervical cancer in a “Screen-and-Treat” program integrated with HIV/AIDS care in Zambia. J Acquir Immune Defic Syndr.

[22] Mboumba Bouassa RS, Prazuck T (2017). Cervical cancer in sub-Saharan Africa: an emerging and preventable disease associated with oncogenic human papillomavirus. Med Sante Trop.

[23] Rohner E, Bütikofer L, Schmidlin K (2020). Cervical cancer risk in women living with HIV across four continents: a multicohort study. Int J Cancer.

[24] Ghebre RG, Grover S, Xu MJ (2017). Cervical cancer control in HIV-infected women: past, present and future. Gynecol Oncol Rep.

[25] WHO (2020). Global strategy to accelerate the elimination of cervical cancer as a public health problem and its associated goals and targets for the period 2020 – 2030.

[26] Phaswana-Mafuya N, Peltzer K (2018). Breast and cervical cancer screening prevalence and associated factors among women in the South African general population. Asian Pac J Cancer Prev.

[27] Godfrey MAL, Mathenjwa S, Mayat N (2019). Rural Zulu women’s knowledge of and attitudes towards Pap smears and adherence to cervical screening. African J Prim Heal Care Fam Med.

[28] Maree JE, Moitse KA (2014). Exploration of knowledge of cervical cancer and cervical cancer screening amongst HIV-positive women. Curationis.

[29] Bradley J, Risi L, Denny L (2010). Widening the cervical cancer screening net in a South African Township: who are the underserved?. Health Care Women Int.

[30] Shisana O, Rhele T, Simbayi LC (2014). South African National HIV Prevalence, Incidence and Behaviour Survey, 2012.

[31] Jooste S, Mabaso M, Taylor M (2020). Trends and determinants of ever having tested for HIV among youth and adults in South Africa from 2005–2017: results from four repeated cross-sectional nationally representative household-based HIV prevalence, incidence, and behaviour surveys. PLoS One.

[32] Tafadzwa D, Julien R, Lina B (2020). Spatiotemporal modelling and mapping of cervical cancer incidence among HIV positive women in South Africa: a nationwide study. Int J Health Geogr.

[33] Croxford S, Kitching A, Desai S (2017). Mortality and causes of death in people diagnosed with HIV in the era of highly active antiretroviral therapy compared with the general population: an analysis of a national observational cohort. Lancet Public Health.

[34] Eyawo O, Franco-Villalobos C, Hull MW (2017). Changes in mortality rates and causes of death in a population-based cohort of persons living with and without HIV from 1996 to 2012. BMC Infect Dis.

